# Enhancing aerobic composting of food waste by adding hydrolytically active microorganisms

**DOI:** 10.3389/fmicb.2024.1487165

**Published:** 2024-12-02

**Authors:** Vladimir Mironov, Vitaly Zhukov, Kristina Efremova, William F. Brinton

**Affiliations:** ^1^Winogradsky Institute of Microbiology, Federal Research Center of Biotechnology, Russian Academy of Sciences, Moscow, Russia; ^2^Woods End Agricultural Institute Inc., Mount Vernon, ME, United States

**Keywords:** hydrolytic microorganisms, composting, anaerobic metabolism, *Bacillus*, *Penicillium*

## Abstract

The biomass of native microorganisms in food waste (FW) suitable for accelerated composting is initially low and requires time for adaptation. Adding of efficient hydrolytic microorganisms should be able to enhance compost-specific microbial activity, adjust microbial community structure, and potentially hasten FW biodegradation. This study aimed to identify bacterial and fungal strains with growth characteristics suitable for accelerating FW composting. Over 7 weeks, FW was composted in a pilot-scale test, either inoculated at the start or on day 28 with three different mixtures of 10 autochthonous *Bacillus* and *Penicillium* spp. strains known for their high hydrolytic activity. The effects of inoculation were assessed by measuring the rate of carbon dioxide (CO_2_) and ammonia (NH_3_) production and also the increase in temperature due to spontaneous exothermic activity of the enhanced microbial population degrading FW. Inoculation with *Bacillus* spp., particularly *B. amyloliquefaciens* and *B. subtilis*, at the beginning of composting increased CO_2_ production nearly 3-fold while maintaining stable ammonia production and temperature. The high concentration of *Bacillus* relative to native FW microorganisms led to dominant fermentation processes even in the presence of oxygen, resulting in moderate heat release and elevated production of volatile organic compounds. Introducing *Penicillium* spp. at a later stage (day 28) increased CO_2_ production nearly 2-fold, along with higher NH_3_ levels and temperature. These findings highlight the significance of inoculation timing and microbial composition in regulating metabolic pathways during FW composting degradation, offering insights for designing effective microbial formulations for composting.

## 1 Introduction

Food waste (FW) is generated at all stages of the food supply chain, from the farms to the ultimate consumers (Parfitt et al., 2010). FW consists of unharvested crops, trimmings and peels, by-products, stale or expired products, damaged goods, unused ingredients, leftovers, and packaging waste. About 1.3 billion tons of FW are generated globally each year, and these amounts are expected to be increasing for the next 25 years (Li et al., 2020). Composting is an efficient way to transform food waste into an organic fertilizer for plant growth, providing a recycling option instead of a one-way process (Wang et al., 2024).

FW are characterized by heterogeneous and variable composition and high moisture content of 65–80% (Awasthi et al., 2017; Cerda et al., 2018). The biomass of intrinsic composting-specific FW microorganisms is low in the early stages of composting and it takes some time for these organisms to proliferate (Xi et al., 2015). Lactic acid microorganisms of the order *Lactobacillales* predominate in the initial FW (Mironov et al., 2021). These bacteria consume readily available organic compounds (primarily sugars) by fermentation and release organic acids, which leads to a decrease in substrate pH and slower microbial degradation (Brinton, 1998; Wang et al., 2024). In addition, even at high temperature (60°C), pathogenic mesophilic microorganisms *Salmonella* sp., *Pseudomonas aeruginosa* have been found in the active phase of FW composting (Droffner et al., 1995). Therefore, processing of FW remains a challenge.

Artificial addition of ‘IM − introduced microorganisms’ can rapidly adjust the microbiota structure and improve microbial activity (Fang et al., 2019; Li et al., 2020). The use of IM reduces the initial acidification of FW (Song et al., 2018; Nakasaki et al., 2013; Nakasaki and Hirai, 2017), shortens composting time (Wang et al., 2024; Barrena et al., 2006), waste volume (Duan et al., 2020; Cerda et al., 2018) and malodour emissions (Fan et al., 2018; Liu et al., 2024), and improves the quality of the finished product (Voběrková et al., 2017; Ballardo et al., 2017).

As IM for FW composting, bacteria of genera are most often used *Bacillus* (Song et al., 2018; Awasthi et al., 2017; Duan et al., 2020; Wang et al., 2024; Zhang et al., 2024), *Geobacillus* (Sarkar et al., 2010; Fang et al., 2019), as well as fungi of the genera *Trichoderma*, *Aspergillus* (Awasthi et al., 2014; Heidarzadeh et al., 2019) and white-rot fungi (Voběrková et al., 2017). The use of microorganisms isolated from composted waste and most adapted to the conditions is promising (Sarkar et al., 2010; Awasthi et al., 2017). For example, representatives of the genera *Bacillus*, *Caldibacillus*, *Aspergillus* and *Penicillium* were the dominant degraders in the FW aerobic composting (Li et al., 2020; Mironov et al., 2021).

The concentration of IM in the waste is usually brought to values of ∼10^5^–10^8^ CFU g^−1^ FW (Ke et al., 2010; Song et al., 2018). Probably, these values depend on the type of IM. Thus, a concentration of ∼10^5^ CFU g^−1^ was used for actinomycete of the genus *Thermoactinomyces* (Ke et al., 2010), fungal compositions of the genera *Trichoderma* and *Aspergillus* (Awasthi et al., 2014), and bacterial-fungal compositions (Xi et al., 2015). For mesophilic the acid-consuming yeast of the genus *Pichia*, a concentration of 10^7^ CFU g^−1^ was used (Nakasaki et al., 2013). For bacterial inoculants (mainly members of the genus *Bacillus*), concentrations of 10^6^–10^7^ CFU g^−1^ were used for isolated cultures (Zhang et al., 2024; Rastogi et al., 2019; Ballardo et al., 2017) and 10^8^ CFU g^−1^ for consortia (Song et al., 2018). The concentration of autochthonous microbiota in source wastes before composting can be 10^6^–10^7^ CFU g^−1^ and 10^9^ CFU g^−1^ during the period of highest decomposition rate (Awasthi et al., 2014; Manu et al., 2017; Mironov et al., 2021). Probably, the effect of IM depends on the quantitative and qualitative composition of the waste’s own microbiota.

IM inoculation is mainly performed at the start of composting (Xie et al., 2023; Awasthi et al., 2017), but there are also reports of inoculation at the end of the thermophilic stage (Voběrková et al., 2017) and in several stages (Zhu et al., 2023; Xi et al., 2015). Probably, multistage inoculation can be more effective than single-stage inoculation due to the possibility of using specific microorganisms adapted to the current stage of the process (availability of substances, temperature, pH, oxygen availability). And it also avoids the competition of the inoculum with the waste’s own microorganisms (Xi et al., 2015). Thus, despite the existing research results, the issues of screening microorganisms to enhance aerobic composting of FW are relevant. There is still insufficient information on how the timing of inoculation and the quantitative ratio between the introduced and autochthonous microbiota affect FW degradation.

IM enhance composting either directly through the activity of the inoculum cultures themselves or indirectly by influencing their own waste microbiota. Wang et al. (2024) conclude that inoculum had a significant effect on the succession of the bacterial community, which caused the enhancement of composting. Inoculation can improve the community structure and functional diversity of microorganisms and enhance the growth of dominant microorganisms (Xi et al., 2015). Analyzing the succession of FW microbial community under the influence of IM can provide insight into the mechanisms of inoculant action.

In the absence of a standardized methodology for evaluating the action of IM in composting, it is difficult to assess the effect. It is useful to express the response to inoculation of microorganisms by indicators that are well suited to instrumental evaluation, e.g., self-heating temperature, CO_2_ and ammonia production dynamics. The measurement of CO_2_ production quantifies the degradation of organic matter during composting (Sánchez-García et al., 2015). This indicates progressive stabilization of the material, as CO_2_ production is often used as a respirometric index that reflects microbial activity. Most NH_3_ emissions are associated with ammonification, which can reveal the rate of decomposition of organic nitrogen, such as protein (Wang et al., 2024). In turn, temperature shows the rate of organic matter degradation and is related to microbial metabolic activity during composting.

Thus, this study aimed to screen active autochthonous microorganisms for rapid decomposition of raw FW under controlled aerobic composting conditions. The effect of inoculation was evaluated on the basis of data on the dynamics of CO_2_, ammonia production and temperature change. Experimental studies were carried out on non-sterile raw FW under pilot-scale test composting conditions.

## 2 Materials and methods

### 2.1 Creating compositions of FW-degrading microorganisms

Our strategy of stepwise screening for potential FW degraders included the following steps (Supplementary Figure 1): (1) obtaining biological material from composted FW; (2) obtaining parent and pure cultures of microorganisms; (3) testing their metabolic activity; (4) composing microbial formulations and testing the cultures for antagonism; (5) evaluating the effects of inoculation during FW composting in a pilot-scale test, and (6) investigating the mechanism of action of the effective composting composition.

#### 2.1.1 Obtaining enrichment cultures and isolating pure cultures

To obtain enrichment cultures of microorganisms, samples of composted waste (food waste, wood chips, excess sludge from biological treatment facilities of dairy production, and mechanically sorted organic fraction of solid municipal waste) were collected at the “Grunt Eco” industrial composting plant (Moscow oblast’; 55°32′26″N, 38°4′46″E).

For microbiological analysis, 1 g samples of composted waste were resuspended in 10 mL of sterile tap water with 5-mm glass beads (Sigmund Linder GmbH, Germany) and shaken for 30 s on Bio Vortex V1 (Biosan, Latvia). Next, series of 10-fold dilutions were prepared and plated on reinforced clostridial agar (g L^−1^): yeast extract 13.0, peptone 10.0, NaCl 5.0, sodium acetate 3.0, glucose 5.0, starch 1.0, cysteine hydrochloride 0.5; pH 7.0 and incubated for 3–4 days at temperatures of 28 and 55°C. The choice of the medium was determined by its composition resembling FW in the representation of various organic compounds. The same medium was used to isolate pure microbial cultures from the enrichments.

The isolated pure cultures were tested for their ability to utilize a variety of carbon compounds: starch, milk proteins, cellulose, and lipids. A similar method for the isolation and selection of cultures for composting compositions was described by Kausar et al. (2011) and Awasthi et al. (2017). For cultures growing on various substrates, the ratio of the hydrolysis zone diameter to the colony diameter was used as an estimate of the culture’s substrate-degrading activity (Hankin et al., 1971; Hankin and Anagnostakis, 1975). Specifically, proteolytic activity was determined on modified Eikman agar of the following composition (g L^−1^): peptone 5.0, yeast extract 3.0, skimmed milk powder 1.0, agar 15.0; pH 7.0. Amylolytic activity was assessed on the Starch–mineral salt–agar medium described by Kausar et al. (2011) (g L^−1^): soluble starch 10.0, K_2_HPO_4_ 1.0, MgSO_4_ 1.0, NaCl 1.0, (NH_4_)_2_SO_4_ 2.0, CaCO_3_ 2.0, FeSO_4_ 0.001, MnCl_2_ 0.001, agar 20.0; pH 7.0. The zones of starch hydrolysis were visualized using iodine solution (5% alcohol solution diluted 10 times with water). Cellulolytic activity was determined using a modified Hetchinson–Clayton medium (g L^−1^): carboxymethylcellulose 2.0, NaNO_3_ 2.5, (NH_4_)_2_SO_4_ 2.0, K_2_HPO_4_ 1.0, MgSO_4_·7H_2_O 0.3, NaCl 0.1, CaCl_2_·4H_2_O 0.1, FeCl_3_·6H_2_O 0.01, agar 17.0; рН 7.2. Cellulose degradation was visualized using a 0.1% (w/w) aqueous Congo red solution. Lipolytic activity was determined using a medium with the following composition (g L^−1^): Tween-80 10.0, peptone 10.0; NaCl 5.0, CaCl_2_·2H_2_O 0.1, agar 20.0. The results were analyzed by measuring the diameter of an opaque zone of calcium salts of fatty acids released from Tween-80 around the colonies.

#### 2.1.2 Identification of compost-associated microorganisms

Bacterial and fungal isolates were identified based on 16S rRNA and ITS gene sequences. For this purpose, the microorganisms were pre-grown in 50 mL vials in liquid LB. Once the desired culture density was reached, the culture was washed from the medium and concentrated in Eppendorf microcentrifuge tubes. DNA was isolated from biomass as described in Boulygina et al. (2002). The concentration of the resulting DNA preparations was 30–50 μg mL^−1^. RNA was present in trace amounts (less than 1% by electrophoretic analysis). For each of the samples studied, PCR and subsequent sequencing of the amplified 16S rRNA gene fragments were performed using universal primer systems to detect both eubacteria (11f–1492r) (Lane, 1991) and fungi (ITS5) (White et al., 1990). PCR products were analyzed by electrophoresis in 2% agarose gel at an electric field strength of 6 V cm^−1^. PCR products were isolated and purified from fusible agarose using WizardPCRPreps reagent kit (Promega, USA) according to the manufacturer’s recommendations. Sequencing of the obtained PCR gene fragments was performed according to the Sanger method (Sanger et al., 1977) on an ABI 3730 (3500XL) genetic analyzer (AppliedBiosystems, Inc., USA) using BigDyeTerminatorv.3.1 reagent set (AppliedBiosystems, Inc., USA) at the Core Facility “Bioengineering” of the Research Center of Biotechnology RAS. The primary similarity analysis of the obtained nucleotide sequences was performed using the BLAST software package (Camacho et al., 2009).

#### 2.1.3 Preparation of bacterial and fungal inocula

Microbial cultures were selected for inoculum compositions based on their highest hydrolytic activity, and their main morphological features (colony and cell morphology) and physiological characteristics (temperature range of growth) were described.

To avoid antagonism between strains included in the same composition, their mutual influence was assessed using the cross-streak method (Egorov, 2004; Lertcanawanichakul and Sawangnop, 2008). The first culture was seeded with a streak on the agar surface in a Petri dish. After the colonies appeared, the second culture was seeded perpendicularly to the streak, and the dishes were placed in a thermostat at 28°C for 24 h. Culture inhibition was recorded if the colony size decreased in comparison to normal growth of that culture.

To prepare inoculum compositions, the selected bacterial cultures were first grown separately in 150 mL of liquid LB medium (g L^−1^: triptone 10.0, yeast extract 5.0, NaCl 5.0, agar 15.0) at 28°C and 130 rpm for 2 days. The cultures were washed from the medium, resuspended in sterile tap water, and mixed in equal amounts (v/v) as described by Awasthi et al. (2017). The resulting inoculum (300 mL) was added to the composting substrate with a volume of 10 L and an average wet sample weight of 4,500 g (average dry matter weight, 1,230 g; average organic matter weight, 1,070 g). To assess the effect of inoculation dose, suspensions with cell titers of 10^6^ CFU mL^−1^ and 10^9^ CFU mL^−1^ of each culture were used. The total concentration of cells added to composting substrate was 4 × 10^5^ CFU and 1 × 10^8^ CFU g^−1^ raw weight FW of the original moisture content. The control substrate was supplemented with 300 mL of tap water. The substrate was thoroughly mixed to ensure uniform distribution of the inoculum.

To obtain the inoculum of the fungal composition, the selected fungi were cultivated on Czapek agar (g L^−1^: sucrose 30.0, NaNO_3_ 3.0, K_2_HPO_4_ 1.0, MgSO_4_·7H_2_О 0.5, FeSO_4_·7H_2_O 0.01, agar 15.0) until a mycelium with spores appeared. After 7 days of cultivation, fungal spores were collected from Petri dishes and suspended in sterile water to a concentration of 10^6^ CFU mL^−1^. The inoculum of the fungal composition was prepared by mixing the spore suspensions and added to FW to obtain a final spore concentration of 10^4^ CFU g^−1^ raw weight FW.

### 2.2 Effects of inoculation on FW composting in a pilot-scale test

#### 2.2.1 General design

Experimental composting of non-sterile food waste was carried out in 2 variants. In option I (START), raw food waste was inoculated and then composted for 28 days. Uninoculated raw food waste was composted as a control experiment. In option II (RESTART), pre-composted food waste from the control experiment after 28 days was supplemented with tap water to an optimum moisture content of about 60% (wt/wt) and air-cooled for 1–2 h to ambient temperature so as to equalize the experimental conditions. The substrate thus obtained was inoculated and composted for 21 days. The experiments were conducted under laboratory conditions at a temperature of 22.1 ± 2.8°C and a humidity of 32.2 ± 4.1%.

The inoculum compositions were separately applied at 2 stages of composting: START or RESTART.

The effect of the inoculum compositions on the composting efficiency was assessed based on CO_2_ formation (principal criterion), NH_3_ formation, and composting temperature T_av_ (auxiliary criteria). Criteria: (i) the average CO_2_ formation during the composting period should be greater than in the control without inoculation (Test >Control); (ii) the average NH_3_ formation during the composting period must be greater than or equal to the control without inoculation (Test ≥Control); (iii) mean temperature T_av_ should be greater than or equal to the mean temperature in the control without inoculation (Test ≥Control). The results were graded as “+” if the average values of these parameters were higher in the experiment than in the control or equal; overwise, they were graded as “–.” If the difference between the experimental and the control values was more than 2-fold or more than 5°C, the result was graded as significant: “++” (or “– –”).

#### 2.2.2 Composting substrate

FW substrate obtained in a waste processing facility consisted of expired products in typical proportions (wt%): potato, 18.0; cabbage, 18.0; apple, 7.2; orange, 7.2; banana, 7.2; minced meat, 3.6; fish, 1.4; bread, 7.2; cottage cheese, 1.4; and chicken egg, 0.7. The solid filler materials included (wt%) waste paper, 11.0; plastic, 10.6; textiles, 0.7; and wood chips, 5.8. The components were homogenized into fragment of ≤10 mm on an IK-07E fodder shredder (Avtomash, Russia) and mixed thoroughly in a BSE-63 concrete mixing machine (Kalibr, Russia). For each series of experiments, 80 L of solid raw FW were prepared as the substrate for the START experiment. The physicochemical parameters of the substrates (analyzed as described in Section 2.2.3) are presented in Table 1.

**Table 1:** The physicochemical parameters of FW (mean ± SD).

Parameters	Units	FW (START)	Pre-composted FW (RESTART)
pH	pH units	6.5 ± 0.1	7.2 ± 0.4
Moisture content	wt%	72.6 ± 1.2	60.7 ± 1.1
Organic matter (OM)	%	87.2 ± 1.0	77.3 ± 0.3
C/N ratio	–	44.9 ± 1.8	41.3 ± 0.7

#### 2.2.3 Test system

**Figure 1:**
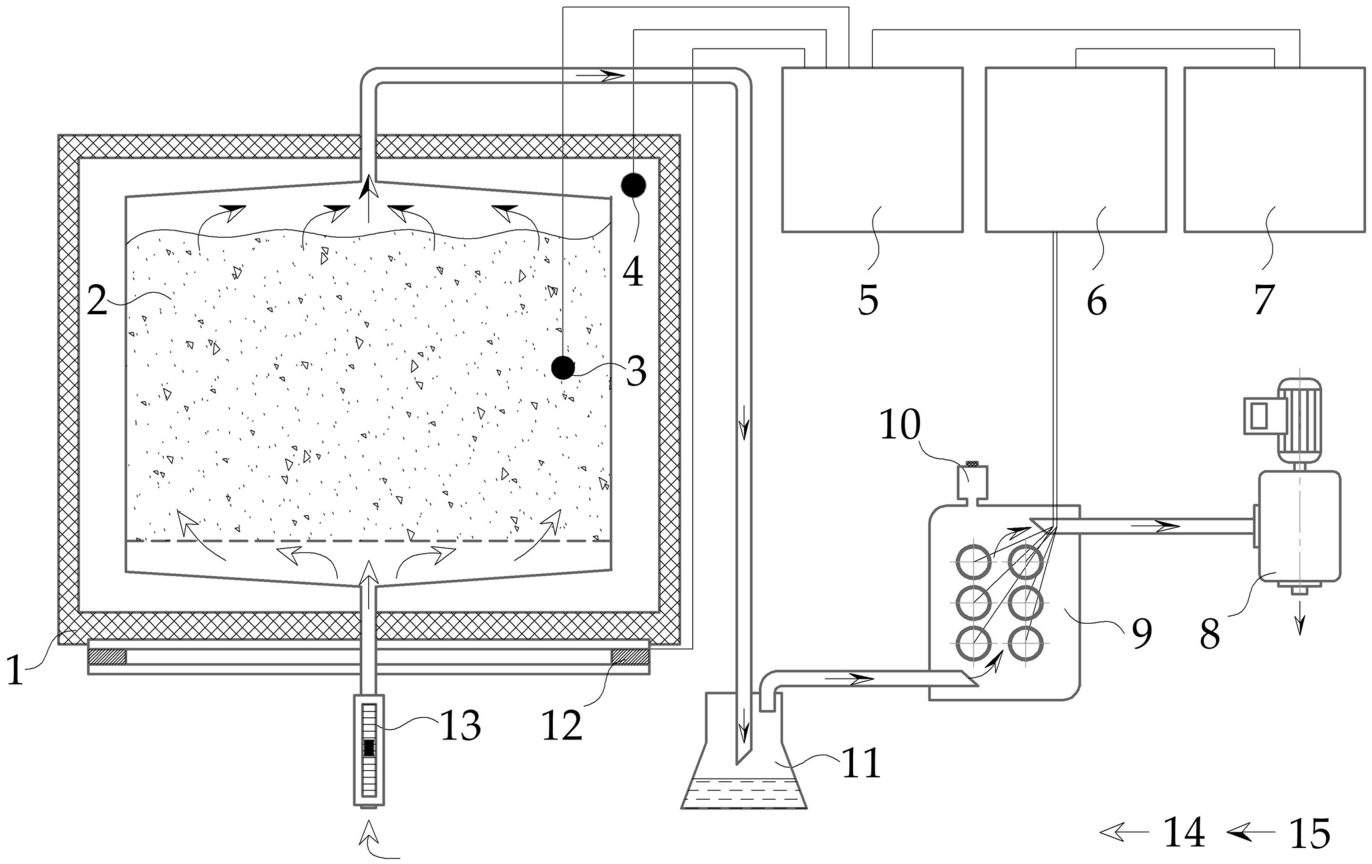
Schematic image of a test (laboratory) setup simulating the conditions of aerobic solid phase biodegradation: chamber (1); reaction vessel (2); temperature sensors (3, 4); temperature meter-regulator (5); gas analyzer (6); computer (7); blower (8); gas sensors (9); sampling port for solid phase micro extraction (SPME) (10); flask (11); heating element (12); rotameter (13); atmospheric air (14); waste air (15).

Composting was carried out in the pilot-scale test system for 49 days in 8 identical chambers according to the scheme (Figure 1) with a total volume of 200 dm^3^. For each variant, 2 reaction vessels of 10 dm^3^ each were designed simultaneously. The test system fully reproduced the conditions of spontaneous temperature change under control of aeration regime and heat loss compensation. The design and performance characteristics of the pilot-scale test system were in accordance with Mironov et al. (2024). The number of repetitions of each variant of the experiments was a minimum of 2 and a maximum of 8 (in the case of the best composition). The dynamics of the biological and physicochemical parameters of the substrate: temperature (T), moisture content, рН, total microbial count (TMC, CFU), germination index (GI), C/N ratio, and organic matter (OM), were analyzed as described in work (Mironov et al., 2021). The content of CO_2_, NH_3_, CH_4_, and H_2_S was analyzed with a MAG-6C-1 gas analyzer (Eksis, Russia), and volatile organic compounds (VOCs) were analyzed by chromatography combined with vapor preconcentration using the solid phase microextraction (SPME) technique as described (Mironov et al., 2023).

To calculate TMC, series of 10-fold dilutions were seeded on modified LB agar (g L^−1^): yeast extract 2.0, peptone 5.0, glucose 1.0, NaCl 1.0, tap water; рН 7.0. The Petri dishes were incubated for 3–4 days at 30 and 55°C. TMC values were obtained by counting the colonies and converting the results to colony forming units per 1 g of substrate (CFU g^−1^).

#### 2.2.4 Community profiling

For NGS profiling of the microbial community, samples were taken from the reaction vessels inoculated with the effective composition and the uninoculated control at the beginning of composting (initial FW community) and on day 15 of the process. Due to substrate heterogeneity, the material was taken from different parts of the reaction vessel into a sterile Falcon tube and homogenized manually. The composition of the bacterial community was analyzed based on the abundance of 16S rRNA genes. DNA isolation, PCR, and Illumina MiSeq high-throughput sequencing of the 16S rRNA gene region were performed using conventional techniques as described (Mironov et al., 2021). For each sample, DNA isolation was performed without replication; for each DNA sample, library preparation and sequencing were performed in duplicate. All 16S rRNA gene sequence reads were then processed using the SILVAngs 1.3 pipeline (Quast et al., 2013) with default settings: 98% similarity threshold was used to generate operational taxonomic unit (OTU) tables; the minimum similarity to the nearest relative used for classification was 93%.

### 2.3 Statistical analysis

To evaluate the effectiveness of the inoculation, the dynamics of the principal (CO_2_ formation) and auxiliary (T_av_ and NH_3_ formation) parameters was monitored for 28 days of START and 21 days of RESTART experiments. Gas composition was determined on days 0–1, 4–8, 11–15, 18–22, and 25–28 for START and on days 0–1, 4–8, 11–15, 18–21 for RESTART. All data were the means from at least 2 parallel experiments. Moisture content, pH, OM, C/N ratio, VOCs, and TMC were measured in 2 replicates for each sample. For NGS profiling of the microbial community, samples were collected from 2 parallel experiments. Community composition was determined in four samples of each substrate from 2 parallel experiments.

The distributions of the experimental data on the dynamics of the principal (CO_2_ emission) and auxiliary (T_av_ and NH_3_ emission) criteria were tested for normality using the Shapiro–Wilk test (significance level, 0.05). It was found that most data did not a have a normal distribution. Therefore, the Kruskal–Wallis test (KWt) for 2 or more samples was used as a nonparametric method (Ruxton and Beauchamp, 2008; Chen et al., 2023). For KWt, the null hypothesis is stochastic homogeneity and the alternative hypothesis is stochastic heterogeneity (Ruxton and Beauchamp, 2008). The following assumptions were made: variables are continuous and independent after randomization, distributions in each group have a similar shape.

Results were presented or as means and standard errors of the mean (SE) for CO_2_, NH_3_ and T_av_ and as means and standard deviations (SD) for all other data. Statistical analysis of the data was performed with MS Excel (Microsoft, USA).

## 3 Results

### 3.1 Physiological and biochemical characteristics of hydrolytic microorganisms

Altogether, 75 pure cultures of microorganisms were isolated from the composted waste, including 66 bacterial and 9 fungal cultures. Among them, 26 cultures showed the ability to hydrolyze one or more biopolymers (starch, cellulose, proteins) or lipids. The 10 cultures with the highest hydrolytic activity were selected for 2 bacterial compositions (**B1** and **B2**) and the fungal composition **F** (Table 2).

**Table 2:** Characterization and metabolic activity of isolated cultures during degradation of different substrates: ratio between hydrolysis zone diameter and colony diameter (mean ± SD).

Culture (GenBank accession number)	Name	Metabolic activities				Morphology of colonies	Cell morphology
		Proteolytic	Amylolytic	Lipolytic	ceLlulolytic		
*Bacillus subtilis* BS2022 (PRJNA979896)	**B1**, **B2**	2.18 ± 0.40	1.91 ± 0.77	unmanifested	1.79 ± 0.76	Off-white, glossy, flat, granular, irregular in shape	Motile short bacilli, streptobacilli, form spores
*Bacillus inaquosorum* GMPCOW1 (OR166016)	**B1**	1.25 ± 0.05	2.58 ± 0.63	0.81 ± 0.17	1.83 ± 0.49	Off-white, glossy, flat, granular, irregular in shape	Motile short, medium-length bacilli and chains, there are spores and cells with endospores located centrally and without bloating
*Bacillus spizizenii* GMPCOW4 (OR187152)	**B1**	1.60 ± 0.35	1.44 ± 0.51	1.19 ± 0.32	2.76 ± 0.92	Matte, transparent, flat, irregular in shape	Motile short bacilli, some are in medium-length and long, no spores
*Bacillus xiamenensis* GMPCOW3 (OR187099)	**B1**	1.36 ± 0.06	2.30 ± 0.17	unmanifested	1.79 ± 0.26	White, matte, flat, irregular in shape, heterogeneous	Motile average size bacilli, diplobacilli, form spores
*Bacillus velezensis* GMPCOW2 (OR185556)	**B1**	1.98 ± 0.39	1.50 ± 0.17	unmanifested	2.24 ± 0.42	Off-white, matte, flat, irregular in shape	Motile bacilli, medium-length, form short streptobacilli and diplobacilli, in some cells there are laterally located endospores without bloating
*Bacillus amyloliquefaciens* BAM2022 (PRJNA979896)	**B2**	1.54 ± 0.13	1.83 ± 0.52	unmanifested	1.57 ± 0.58	Off-white, glossy, folded, round	Motile medium-length bacilli, form spores
*Penicillium* sp. strain GMPCOW5 (OR187303)	**F**	1.95 ± 0.28	1.96 ± 0.18	1.58 ± 0.32	1.65 ± 0.18	White-green colonies are convex, matte, filamentous, round	Mycelium
*Penicillium* sp. strain GMPCOW6 (OR187306)	**F**	1.21 ± 0.19	1.73 ± 0.09	1.57 ± 0.25	1.07 ± 0.12		
*Penicillium crustosum* + *Penicillium chrysogenum* GMPCOWM1 (PRJNA997142)	**F**	1.20 ± 0.06	1.58 ± 0.14	2.01 ± 0.28	1.73 ± 0.18		

Composition **B1** included five cultures of the genus *Bacillus*. It has been repeatedly observed that *Bacillus* members dominate among easily cultivated microorganisms of composted waste (Blanc et al., 1997; Mironov et al., 2021). These cultures were isolated at the high-temperature stage of composting (45–60°C), but grew actively at 30°C, which indicated their thermotolerance (Moreno et al., 2021). Bacteria of composition **B1** were capable of degrading proteins, starch, cellulose, and partially lipids.

**Figure 2:**
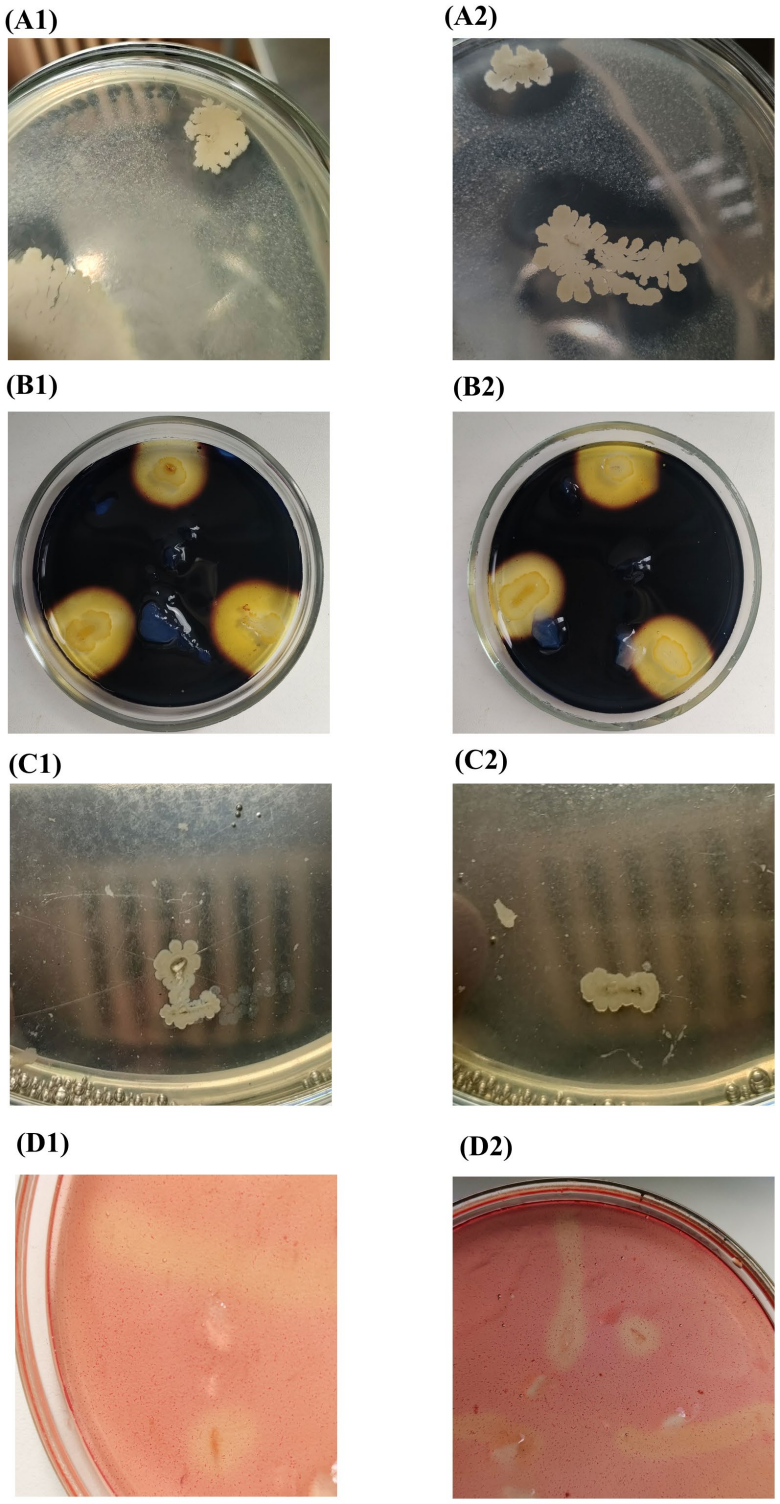
Metabolic activity of microorganisms (1) *Bacillus subtilis* and (2) *Bacillus amyloliquefaciens* of composition **B2**: **(А)** proteolytic; **(B)** amylolytic; **(C)** lipolytic; **(D)** cellulolytic.

Composition **B2** was prepared from 2 cultures: *Bacillus subtilis* and *Bacillus amyloliquefaciens*. The temperature range of their growth was 20–55°C. Both cultures were capable of hydrolyzing starch and cellulose (Figure 2), but differed in their ability to utilize milk proteins as the only carbon source. In addition, both cultures were incapable of lipid hydrolysis (Figure 2C). Previously, it was found that *Bacillus* cultures can grow under anaerobic and microaerophilic conditions through mixed acid fermentation (with formation of acetate, lactate, formate, CO_2_ and other compounds) and anaerobic respiration (nitrate and fumarate) (Hoffmann et al., 1995; Clements et al., 2002).

Fungal composition **F** included four cultures of the genus *Penicillium*; 2 cultures were identified at the genus level, and the other 2 were a mix of *Penicillium crustosum* and *Penicillium chrysogenum*. These cultures were isolated at high-temperature composting conditions (45–60°C). Under laboratory conditions, they grew at 20–35°C and actively degraded lipids (Tween–80), starch, cellulose, and proteins.

**Figure 3:**
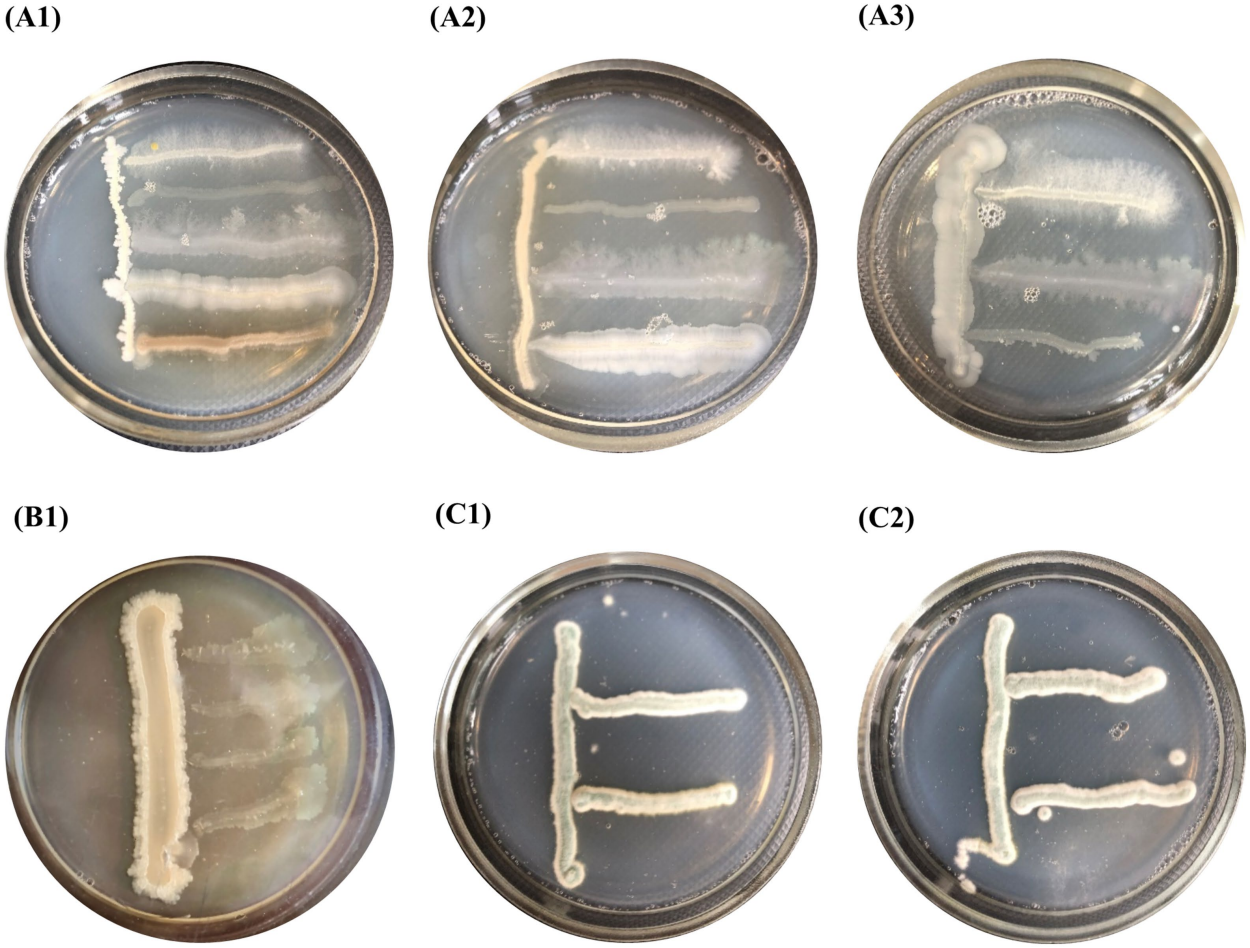
Compatibility of pure cultures shown by the perpendicular stroke method: **(A1–A3)** composition **B1**, **(B1)** composition **B2** and **(C1–C2)** composition **F**.

Using the cross-streak method, it was shown that the cultures included in compositions **B1**, **B2**, and **F** did not inhibit each other’s growth. The colony size of the test cultures did not decrease compared to their normal growth (Figure 3).

### 3.2 Evaluation of compositions and time of inoculation

The results of evaluation of different compositions based on the principal (CO_2_) and auxiliary (NH_3_, and T_av_) criteria are summarized in Table 3.

**Table 3:** Comparative evaluation of the criteria based on the mass of CO_2_ and NH_3_ and the temperature of self-heating T_av_ during composting (mean ± SE).

Inoculum type	Principal criterion			Auxiliary criterion (1)			Auxiliary criterion (2)		
	CO_2_, mg day^−1^ kg^−1^ OM	KWt	Test > control	NH_3_, μg day^−1^ kg^−1^ OM	KWt	Test ≥ control	Т_av_, °С	KWt	Test ≥ control
START									
B1* control	184 ± 30	9.81 > *H*_crit_	– –	150 ± 55	1.68 < *H*_crit_	+	50.5 ± 2.1	0.72 < *H*_crit_	+
	735 ± 154			270 ± 58			47.5 ± 2.4		
**B1** control	891 ± 128	1.43 < *H*_crit_	−	242 ± 69	0.01 < *H*_crit_	+	42.7 ± 1.5	17.96 > *H*_crit_	– –
	669 ± 130			228 ± 62			53.3 ± 1.8		
**B2** control	1778 ± 319	8.30 > *H*_crit_	++	251 ± 57	0.16 < *H*_crit_	+	52.3 ± 2.0	0.19 < *H*_crit_	+
	644 ± 122			242 ± 50			51.4 ± 1.9		
**F** control	314 ± 59	5.13 > *H*_crit_	– –	439 ± 93	1.58 < *H*_crit_	+	43.7 ± 1.6	3.81 < *H*_crit_	+
	735 ± 154			270 ± 58			47.5 ± 2.4		
RESTART									
**B1** control	368 ± 182	2.39 < *H*_crit_	−	144 ± 18	1.41 < *H*_crit_	+	30.6 ± 2.1	0.01 < *H*_crit_	+
	212 ± 89			181 ± 23			31.9 ± 2.5		
**B2** control	501 ± 228	8.34 > *H*_crit_	++	158 ± 41	0.10 < *H*_crit_	+	31.3 ± 1.4	5.51 > *H*_crit_	−
	208 ± 81			166 ± 38			34.2 ± 1.3		
**F** control	402 ± 75	4.30 > *H*_crit_	+	337 ± 53	7.99 > *H*_crit_	++	43.1 ± 1.5	4.46 > *H*_crit_	++
	228 ± 70			110 ± 53			37.5 ± 2.3		

* – **B1** inoculum with concentration 4×10^5^ CFU g^−1^; implementation «+» and non-implementation «–» of the criterion, or significant implementation «++» or non-implementation «– –» of the criterion in case of exceeding (decreasing) by ≥2 times or ≥5°C, the values of the parameters.

The effect of inoculum concentration was assessed using **B1** suspensions with total cell titers of 4 × 10^5^ and 10^8^ CFU g^−1^ FW, which corresponded to the lowest and highest concentrations previously used in inoculation studies (Barrena et al., 2006; Awasthi et al., 2017; Song et al., 2018; Duan et al., 2020; Rastogi et al., 2019). The dynamics of CO_2_, NH_3_, and T_av_ for the START and RESTART variants are shown in Supplementary Figures 2, 3, respectively.

Introduction of composition **B1** at a low cell concentration (10^5^ CFU g^−1^) led to a decrease in biodegradation intensity: CO_2_ formation in the substrate with **B1** (10^5^) was significantly (four-fold) lower than in the control, with no significant differences in NH_3_ production and T_av_. Apparently, at this concentration, the introduced hydrolytic microorganisms had difficulty competing with the original microbial community of the waste.

The use of composition **B1** with a concentration of 10^8^ CFU g^−1^ in the START variant led to a significant decrease (by 10.6°C) in the average temperature over the entire period, without differences in the formation of CO_2_ and NH_3_.

Based on the principal and auxiliary criteria, composition **B2** consisting of 2 *Bacillus* cultures exhibited significant advantages over **B1** and **F** when applied in the START variant. Inoculation with composition **B2** at 10^8^ CFU g^−1^ resulted in the highest (2.8-fold) increase in CO_2_ formation, while maintaining an acceptable level of NH_3_ formation and the T_av_ values as in the uninoculated control. Inoculation with **B1** and **F** resulted in a significant decrease in T_av_ and CO_2_ production, respectively.

In the RESTART experiments, there was no response to the application of **B1**. In the case of **B2**, CO_2_ production increased by 2.4 times and the autoheating T_av_ decreased by 2.9°C compared to the control without inoculum, while ammonia emission was similar. Inoculation with composition **F** increased CO_2_ emission by 1.8 times, T_av_ by 5.6°C, and NH_3_ emission by 3.1 times compared to the control. Thus, **B2** and **F** showed similar positive results in the RESTART variant.

### 3.3 Dynamics of changes in biological and physicochemical parameters from the action of composition B2

**Figure 4:**
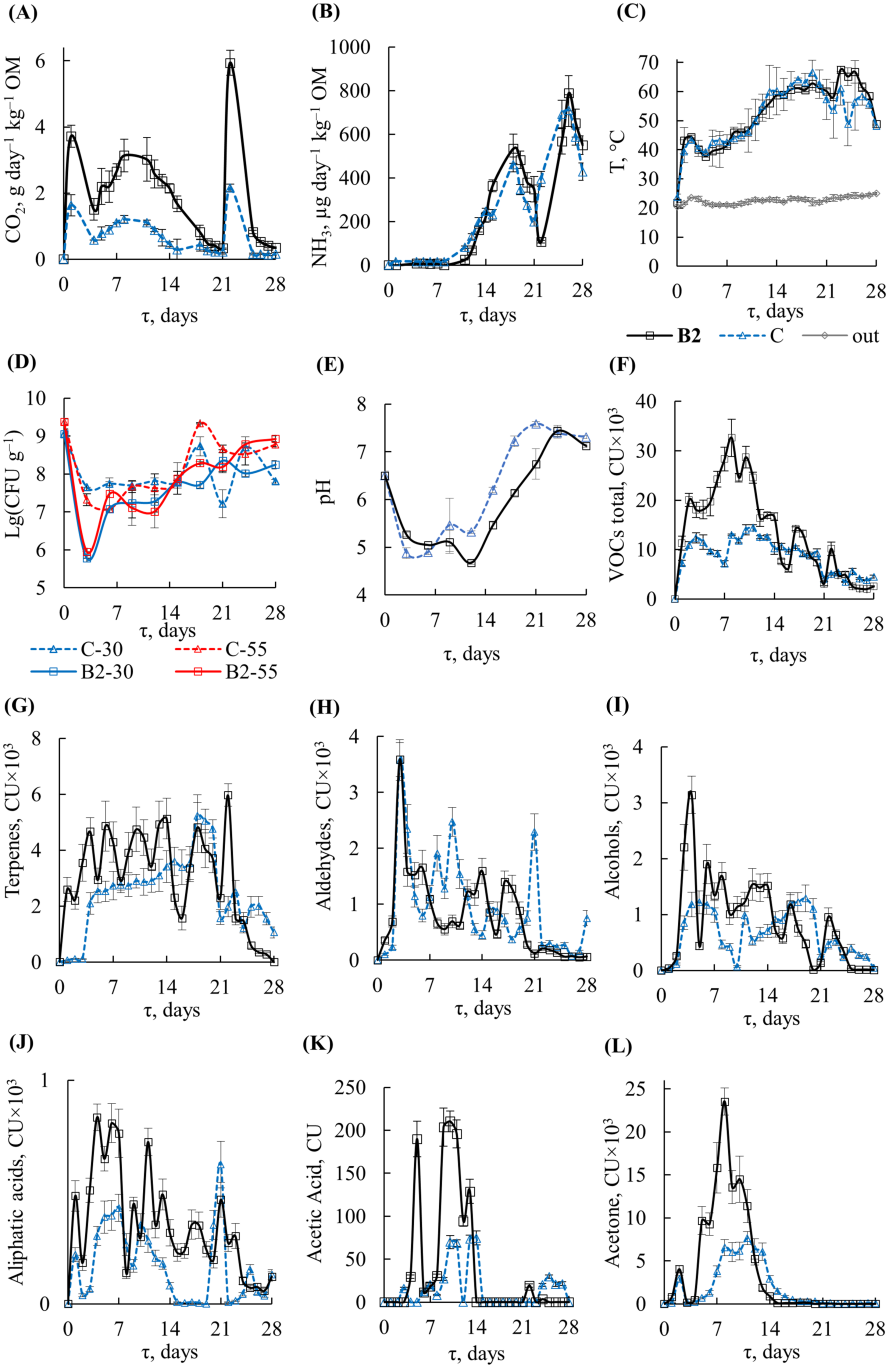
START with **B2**: profiles of **(A)** CO_2_, **(В)** NH_3_, **(С)** temperature, **(D)** TMC, **(E)** pH, **(F)** VOCs total, **(G)** terpenes, **(H)** aldehydes, **(I)** alcohols, **(J)** aliphatic acids, **(K)** acetic acid, **(L)** acetone: *τ*, composting time; **B2**, with inoculation; C, control; out, outside temperature; **B2**-30, С-30, **B2**-55, С-55, TMC for temperatures 30 and 55°C.

**Figure 5:**
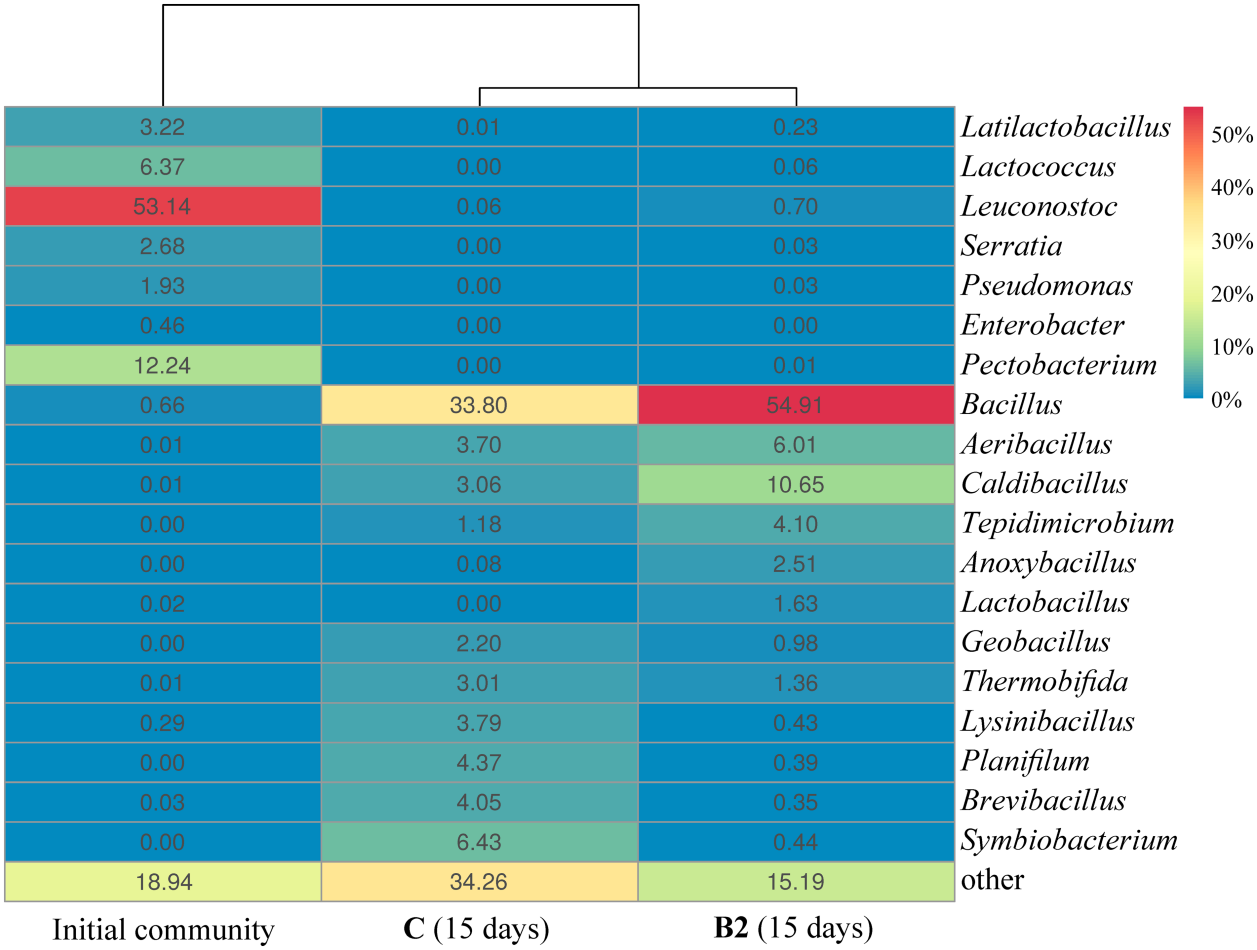
The composition of the bacterial community at the level of genera based on the results of 16S rRNA gene sequencing of microbiota of the substrates (genera representing more than 1% of the community are represented, the remainder are grouped as “other”): Initial community – the initial bacterial community of food waste, С (15 days) and **B2** (15 days) are the bacterial communities of the control and inoculated **B2** substrates, respectively, on day 15 of composting.

Composition **B2** was selected for detailed analysis as the most effective one. Inoculation with **B2** led to changes in the principal composting parameters (Figure 4) and in the succession of the bacterial community (Figure 5).

The dynamics of the principal and auxiliary criteria are presented in Figure 4A–C. TMCs decreased significantly on day 3 by about 1.5 orders of magnitude in the control and by 3.0 orders of magnitude in the inoculated substrate (Figure 4D). The decrease in microbial counts was due to a drastic change in the growth conditions at the start of composting, which led to the death of some species and the promoted the growth of others. The TMC decrease was more pronounced in the inoculated sample, because the supplemented *Bacillus* entered in an active competition with other members of the community, suppressing the development of some groups of microorganisms. Subsequently, TMC values gradually recovered. On days 6–15, TMCs in the control and inoculated substrate remained stable, amounting to 10^7^–10^8^ CFU g^−1^. Under thermophilic conditions, TMCs increased to ~10^9^ CFU g^−1^ by day 28, which was close to the initial numbers in the FW substrate.

The dynamics of pH values after **B2** inoculation and in the control were similar (Figure 4E). The pH decrease to 4.7–5.3 on days 3 and 9 occurred almost simultaneously. The increase in pH was observed on day 12 in the control and on day 15 in the inoculated variant. At the end of the experiment, pH was close to neutral in both variants.

Gas chromatography of the exhaust air detected a total of 121 VOCs. They were represented by 18–29% ketones (acetone, etc.), 23–26% terpenes (D-limonene, *β*-pinene, etc.), 7–10% aldehydes (acetaldehyde, propanal, heptaldehyde, etc.), 6% alcohols (methanol, ethanol, isopropanol), 4% amines (ethylamine, etc.), and 2–3% aliphatic acids (acetic, propionic, butyric, etc.). The highest formation of VOCs in variant **B2** was observed in the first 2 weeks (Figure 4F). In total, 1.6 times more VOCs were detected from the substrate with **B2** inoculum than in the control experiment over a period of 28 days: 390 ± 30 and 251 ± 15 conventional units (CU) × 10^3^, respectively. The maximum VOC formation occurred on day 8 of composting with inoculate **B2** and on day 11 in the control, amounting to 33 ± 4 and 14 ± 1 CU × 10^3^, respectively.

The maximum of terpene emission was detected in the exhaust air from **B2** on day 22 in the control on day 18: 5.9 ± 0.4 and 5.2 ± 0.7 CU × 10^3^, respectively (Figure 4G). The maximum of aldehydes was detected on day 3, and their total emission was nearly independent of the addition of inoculum **B2** (23.1 ± 3.3 and 27.2 ± 4.1 CU × 10^3^) (Figure 4H). Similarly, the formation of amines was not related to the inoculation (for a total of 28 days, 13.5 ± 1.9 vs. 11.1 ± 1.2 CU × 10^3^), with the maximum occurring on day 2.

Acetone (Figure 4L) was detected mainly from day 2 to day 14. Altogether, twice as much acetone was formed in the **B2** variant than in the control over 28 days: 111.9 ± 15.9 vs. 55.3 ± 8.7 CU × 10^3^, respectively. Aliphatic C_2_–C_8_ acids were mainly detected from day 1 to day 14, and their total amount formed over 28 days was 2.1 times higher in the **B2** variant than in the control (10.1 ± 1.3 vs. 4.8 ± 0.8 CU × 10^3^) (Figure 4J). They were mainly represented by acetic, butyric, valerian, and propionic acids. Acetic acid was detected in maximum concentrations from day 5 to day 14 and its total amount in the **B2** variant was 2.5 times higher than in the control (1,136 ± 99 vs. 463 ± 40 CU) (Figure 4K).

Alcohols were represented by methanol, ethanol, and isopropanol and were mainly detected from day 2 to day 14 (Figure 4I). In the **B2** variant, their levels were 34% higher than in the control (total 24.8 ± 3.9 vs. 18.5 ± 3.1 CU × 10^3^).

The formation of CH_4_ reached the highest values on day 1 both in the control of 1.2 ± 0.8 vol. % and with the inoculum of 1.7 ± 1.1 vol. %, with average values for the observation period of 0.23 ± 0.06 and 0.33 ± 0.05 vol. %, respectively. Hydrogen sulfide was detected in the exhaust air at relatively low concentrations in different time periods, averaging 0.06 ± 0.02 mg m^−3^ in the control and 0.29 ± 0.08 mg m^−3^ in the inoculated compost.

The original waste and the composted material obtained after 28 days of incubation with and without inoculum did not inhibit plant growth and development as assessed by GI: it was 73.7 ± 12.4% for original FW, 116.5 ± 21.2% for the control compost on day 28, and 128.2 ± 18.8% for the **B2** compost. OM losses after 28 days of composting were more significant in the substrate with **B2** inoculation (38%) than in the control (28%).

On day 15, the relative abundance of the genus *Bacillus* in FW inoculated with **B2** was 21% higher than in FW without inoculation: 54.9 ± 2.9% and 33.8 ± 1.8%, respectively (Figure 5). The increase in the presence of this genus was probably the result of inoculation and subsequent survival of the introduced microorganisms under the composting conditions.

## 4 Discussion

### 4.1 Effects of composition, inoculation time, and inoculum concentration

The inoculum concentration significantly affected the composting of FW in the START variant. Application of composition **B1** at a higher titer of 10^8^ CFU g^−1^ enhanced biodegradation (assessed by CO_2_ emission) in comparison to using a lower titer. Our results are consistent with those from earlier studies: Fang et al. (2019) reported successful inoculation of raw sludge with thermophilic bacteria of the genus *Geobacillus* with a titer of 10^6^ CFU g^−1^ and Nakasaki et al. (2013) found that inoculation had a significant accelerating effect on composting when the cell concentration was higher than 10^5^ CFU g^−1^. In our study, an inoculum with a low concentration of 4 × 10^5^ CFU g^−1^ had a negative effect on the composting intensity. A similar effect was reported by Ballardo et al. (2017) and Xi et al. (2015), who used multi-stage inoculation to avoid attenuation of the process due to competition. However, Song et al. (2018), managed to accelerate degradation of carbohydrates during FW composting by applying a consortium of autochthonous microorganisms at a concentration of 10^5^ CFU g^−1^. In our study, the proportions between the cell numbers of the inoculated and autochthonous FW microbiota were 1: 2500 and 1: 10 for inoculation using concentrations of 4 × 10^5^ and 10^8^ CFU g^−1^, respectively.

In the START variant, inoculation with the bacterial composition **B1** at 10^8^ CFU g^−1^ or the fungal composition **F** at 10^4^ CFU g^−1^ resulted in a significant decrease in T_av_ in the case of **B1** and CO_2_ formation in the case of F. However, the test for antagonism did not detect any growth suppression effects among cultures of composition **B1**. It is possible that their relationships changed during composting, where the environment is more aggressive, competition is more intense, and various growth factors are limited in comparison to *in vitro* cultivation conditions, which could diminish the effect of the inoculation. A similar effect was observed by Voběrková et al. (2017) and Xie et al. (2023), when the compound microbial agents were inoculated, CO_2_ formation was reduced by 20–30%. The fungal composition F, which consisted of four *Penicillium* members with a pronounced ability to decompose lipids, decelerated FW biodegradation when applied at the beginning of composting. Apparently, these cultures, although isolated under conditions of high-temperature composting, were not active at elevated temperatures during the experiment. It is also possible that the cultures of composition **F** entered into an antagonistic interaction with the autochthonous microbiota of the substrate, which led to a decrease in the biodegradation rate. Similarly, in the study by Heidarzadeh et al. (2019), introduction of *Aspergillus niger* at the beginning of composting negatively affected the loss of total organic carbon and temperature.

Inoculation with composition **B2** at 10^8^ CFU g^−1^ at the START stage augmented the intensity of composting in comparison to the control experiment. Composition **B2** was represented by 2 cultures of the genus *Bacillus* with hydrolytic activity against starch, cellulose, and proteins. A 2.8-fold increase in CO_2_ formation and a 10% increase in OM losses were observed, while NH_3_ formation levels remained acceptable and T_av_ was the same as in the uninoculated control. Our results are consistent with the data from Ke et al. (2010) and Nakasaki et al. (2013): it was shown that inoculation increased CO_2_ formation during composting of both model and real FW. High carbon losses following inoculation with *B. subtilis* were also observed by Duan et al. (2020). Similar results of high titres were obtained by Zhu et al. (2023), Nakasaki and Hirai (2017) and Zhang et al. (2024), who used inoculants at a concentration of 10^7^–10^8^ CFU g^−1^ raw waste.

After 28 days, the substrate with **B2** was by 11% more effective in stimulating germination of the test plants than the control. This was probably due to a decrease in the level of unstable OM, which inhibits germination. The observed increase in GI and OM uptake following microbial inoculation of FW is consistent with data from Manu et al. (2017) and Li et al. (2020).

Following inoculation in the RESTART variant, positive effects of similar magnitude were observed for 2 compositions: bacterial **B2** and fungal F. In the variant with **B2**, CO_2_ emission increased 2.4-fold, but at the same time the average temperature decreased by 2.9°C. Inoculation with composition **F** increased CO_2_ formation 1.8-fold, T_av_ by 5.6°C, and ammonia emission 3.1-fold in comparison to the control. Liu et al. (2024) also observed that a microbial agent stimulated NH_3_ emission at the early stages of composting. A positive effect on waste inoculation with *Bacillus* cultures after a high-temperature period was also shown by Ballardo et al. (2017). If we compare the START and RESTART variants, the hydrolytic bacteria of composition **B2** produced a more pronounced effect when applied at the beginning of the experiment rather than at the cooling stage. Probably, the conditions of pre-composted FW did not correspond to the optimum for their growth, for instance, due to a lower residual content of easily degradable biopolymers (such as starch or pectin). During the 28 days of substrate composting, a considerable portion of easily degradable compounds was consumed, as observed previously (Mironov et al., 2021). The maximum of CO_2_ formation in observed in RESTART, both in the control and with the inoculum, can be related to the mixing of the substrate, which intensified the hydrolysis of the available biopolymers.

Inoculation with the fungal composition **F** at RESTART may be more effective because during the late stage of composting fungi are able to degrade water-insoluble biopolymers, while the amounts of more readily available nutrients in FW become limited. The effectiveness of fungal inoculation at the late stages of composting (cooling and maturation) was also demonstrated by Voběrková et al. (2017) and Xi et al. (2015). The observed increase in the average temperature by 5.6°C in the presence of composition **F** was probably due to the high lipolytic activity of *Penicillium* sp. Similar results were previously reported by Awasthi et al. (2014) for composting with an initial concentration of 10^6^ CFU g^−1^ of autochthonous FW microorganisms. Inoculation with *Aspergillus* and *Trichoderma* fungi in concentrations similar to ours (10^4^ and 10^6^ spores g^−1^) led to a significant increase in temperature and enhanced mineralization of the waste. A comparative evaluation of the effect of inoculation in the composting process is presented in Supplementary Table 1.

### 4.2 Mechanism of action of composition B2

During the first day of composting, substrate degradation occurred via both the pathways that involved O_2_ and those that did not. On the one hand, aerobic degradation probably took place in a liquid film on the surface of solid particles with access to oxygen through air pores. On the other hand, fermentation occurred within solid substrate particles in places inaccessible to oxygen diffusion. The microbial community of FW inoculated with **B2** was diverse and dynamic.

Food waste has a high content of easily degradable organic compounds. Large amounts of available polysaccharides of plant origin can undergo enzymatic hydrolysis. The original microbial community of FW was dominated (>60%) by lactic acid bacteria (LAB) of the genera *Leuconostoc, Lactococcus*, and *Latilactobacillus*. Obviously, on day 1, LAB were active both in the substrate with **B2** and in the control, and the main process was monosaccharide fermentation producing lactic acid, ethanol, and CO_2_.

The observed accumulation of acetic acid under the mixed aerobic-anaerobic conditions was unsurprising, since it is a common product of several bacterial metabolic pathways. In particular, it is produced as a result of mixed acid fermentation by facultatively anaerobic microorganisms, as well as by strictly anaerobic members of the genus *Clostridium*. During FW composting, acetate is mainly derived from pyruvate generated by glycolysis (Song et al., 2018). Under aerobic conditions, acetic acid may be a product of incomplete oxidation of glucose or ethanol, which is transformed to acetaldehyde and then to acetic acid. In our experiment, significant amounts of alcohols, mainly ethanol, were detected in the exhaust air during the first 2 weeks; their level was by 34% higher in the **B2** variant than in the control.

Abundance of easily degradable compounds in the FW substrate together with a high concentration of **B2** inoculum supported intense microbial respiratory activity with moderate heat release and pH decrease during the first 10 days. Significant levels of acetic, butyric, isovaleric, and propionic acids were detected in the exhaust air during this period. According to Nakasaki et al. (2013), acetic, propionic, butyric, and lactic acids are the four dominant organic acids that cause pronounced acidification and slow down the composting process. Song et al. (2018) specified that it may be acetic and propionic acids that cause excessive acidification at the initial stage of FW composting.

In addition to these principal pathways, an alternative pathway may have contributed to acetate formation. Won et al. (2021) showed that acetic acid was a major metabolic product in *Bacillus* spp. growing in the presence of oxygen and with excess of glucose and other easily metabolized carbohydrates. This phenomenon is known as “overflow metabolism” or “the bacterial Crabtree effect”: when glucose uptake in aerobic bacteria exceeds a critical threshold, the carbon flow is directed primarily toward acetate formation. This effect may also occur during composting as a response to substrate excess (Reinhardt, 2002). The phenomenon of overflow metabolism was described for the bacterial consortia of activated sludge flocs from sewage treatment plants, when the specific uptake of glucose exceeded the critical rate corresponding to the maximum respiration rate (Thierie and Penninckx, 2004). Szenk et al. (2017) report the demonstration by *Escherichia coli* of a similar effect: at high growth rates, it switches from respiration to fermentation. Fermentation processes provide the lowest energy production. Probably related to this is the moderate heating of the **B2** substrate with inoculation.

Acetate can also be formed by heterotrophic homoacetogens through carbohydrate fermentation with acetogenesis. Acetate, like other organic acids, is oxidized by microorganisms to carbon dioxide and water. Song et al. (2018) reported that inoculation of FW with a microbial consortium containing members of the genera *Bacillus*, *Lactobacillus*, *Aeribacillus*, and *Pseudomonas* can increase the diversity of microbes producing enzymes that catalyze the reactions of acetic and propionic acid degradation, as well as the abundance of these key enzymes.

A further group of compounds available at the beginning of composting were proteins. The original FW community had a high proteolytic potential, as demonstrated by active ammonification. Protein hydrolysis produced amino acids, which were further degraded to the end products ammonia, carbon dioxide and water.

The totality of aerobic and anaerobic reactions of OM decomposition at the initial stage of FW composting produced a large amount of carbon dioxide: up to 3.73 g CO_2_ day^−1^ kg^−1^ OM, and an equivalent amount of oxygen was required. Due to spontaneous heat release, the temperature increased from 22 to 30°C, which was optimal for the development of LAB; oxygen consumption and CO_2_ formation reached the first local maximum. Inoculation with composition **B2** resulted in a 2.3-fold increase in CO_2_ formation and a 56% increase in VOC formation on day 1. The detected VOCs were mainly aliphatic acids, terpenes, alcohols, and amines.

Jakobsen (1992) observed that CO_2_ and H_2_O formation can be a limiting factor for composting reactions if their excess is not removed from the substrate at the appropriate rate. If all the CO_2_ is removed due to air replacement, the reaction proceeds rapidly.

Apparently, under conditions of high moisture content (72.6 wt%) and O_2_ deficit (~10 vol. %) observed on days 1–3, anaerobic processes accounted for a larger share of OM decomposition reactions, and energy production was moderate. Since the temperature was not high enough, water and CO_2_ were not completely removed from the substrate with the air flow and reacted in the solution to form carbonic acid. As a consequence, only a part of the emitted CO_2_ could be detected in the exhaust air. During this period, the balance between O_2_ absorption and CO_2_ emission was not observed. According to Li et al. (2020), when moisture content exceeds 70%, it is difficult to maintain air circulation inside the reactor and FW tends to produce acidic decay gas as a result of anaerobic fermentation.

The accumulation of CO_2_ prevented the dissolution of oxygen, and microbial activity decreased, while acetic and other organic acids were not completely degraded and probably accumulated in the substrate. By day 3, pH decreased to 4.9–5.3 and TMCs also decreased by 10^3^–10^2^ CFU g^−1^. The decrease was more pronounced in inoculated FW substrates. These results were consistent with observations from previous studies (Sarkar et al., 2010; Mironov et al., 2021).

On days 4 to 6, FW substrates were characterized by relatively low CO_2_ emissions (1.5–2.2 g CO_2_ day^−1^ kg^−1^ ОМ), moderate temperature (39.2–43.1°C), and acidic pH of 5.1. Low pH values due to organic acid formation were reported previously by Awasthi et al. (2017, 2014) and Manu et al. (2017). Similar temperature dynamics was observed without inoculation (Fang et al., 2019) and after inoculation with *Bacillus* spp. at the initial composting stage (Li et al., 2020). During this period, NH_3_ emission from the substrate remained below the detection limit, which could be due to its chemical binding. Ammonia dissolved in water to form ammonium hydroxide or (in the presence of CO_2_) ammonium carbonate. Organic acids reacted with NH_4_OH to form ammonium salts.

During days 7–14, the temperature increased from 40 to 60°C. At the same time, the solubility of CO_2_ and NH_3_ in water decreased 1.5- to 2-fold, which led to a decrease in the formation of ammonium carbonate. In addition, the gradual increase in temperature first caused carbonic acid decomposition into water and CO_2_ and then induced ammonium carbonate decomposition into ammonia and CO_2_. This enhanced the emission of CO_2_ (to its second local maximum) and NH_3_ (to a lower extent) from the substrate. Ammonium hydroxide is more resistant to temperature rise than ammonium carbonate, and its accumulation led to gradual alkalinization of the substrate.

During days 10–12, active decomposition of organic acids and their salts, including ammonium, led to an increase in pH values to 7.4–7.6. Our results are consistent with the data of Reinhardt (2002), who showed that temperatures above 50°C were detected only together with a significant decrease of acetic acid concentrations. Apparently, this is why ammonia was detected in the exhaust air from day 12 onwards. Our results agree with previous observations of microbial activity leading to an increase in pH with growing temperature, volatilization of organic acids, and decomposition of organic nitrogen of FW with formation of ammonium (Li et al., 2020).

Interestingly, while inoculation with **B2** led to a significant 2.8-fold increase in CO_2_ emission, the temperature dynamics did not change, and the pH dynamics was similar to the control, except that the acidic period lasted 3 days longer. Our result differs from the data reported by Wang et al. (2024), who observed an increase in temperature of the inoculated compost. However, their composting experiment was started under thermostated conditions at 50°С. While providing an advantage to thermophilic microorganisms, this protocol deviates considerably from the real-life composting process, which usually begins under normal conditions (18–25°C) and sometimes even at low temperatures of 8–12°С. Nevertheless, the authors concluded that the enhancing in composting was due to an autochthonous microbiota enhanced by an exogenous consortium.

Probably, a slowdown in the decomposition of organic acids and moderate heat generation were consequences of insufficient CO_2_ replacement by O_2_. The high activity of **B2** microorganisms caused oxygen deficiency, which limited the development of aerobes and favored fermentation processes, as evidenced by an increase in the emission of CO_2_, VOCs, CH_4_, and H_2_S without an increase in heat generation. The highest VOC levels in the exhaust air were detected during the first 14–15 days. Furthermore, inoculation with **B2** resulted in a 2.5-fold increase in VOC production. This rise was due to an increase in the content of alcohols, terpenes, aliphatic acids, and ketones (acetone) generated during decomposition of plant components of FW.

During the first 2 weeks, there was a pronounced change in the dominant microorganisms of the compost communities. Apparently, LAB were unable to compete with active development of other bacterial genera, mainly *Bacillus*, which accounted for 54.9% of the total **B2** community by day 15. Quantitatively similar results were obtained by Wang et al. (2024) for FW inoculation with commercial *Bacillus*-containing preparations. In comparison to the control, inoculation with **B2** led to a more pronounced increase in the proportion of *Bacillus* members (54.9 vs. 33.8% in the control by day 15) and promoted the growth of anaerobic hydrolytic bacteria of the genus *Tepidimicrobium* (4.1 vs. 1.18%) and members of *Lactobacillus* (1.63 vs. 0.0%). The replacement of *Leuconostoc* (53.1% of the original community) by *Lactobacillus* was probably related to the increase in temperature to 60°C, which is consistent with the results reported by Fisgativa et al. (2018).

It is known that acetone, ethanol, acetate, propionate, and butyrate can be generated by bacteria, including *Bacillus*, *Caldibacillus*, and *Tepidimicrobium* spp., through fermentation (Hoffmann et al., 1995; Clements et al., 2002). In particular, high levels of hydrolytic activity were demonstrated for strictly anaerobic bacteria of the genus *Tepidimicrobium* and facultatively anaerobic members of *Caldibacillus* (Wushke et al., 2017; Shikata et al., 2018). For example, *Tepidimicrobium ferriphilum* (order *Clostridiales*) is a moderately thermophilic anaerobic bacterium with a growth temperature range of 26–62°C and an optimum of 50°C (Slobodkin et al., 2006). The pH range of its growth is 5.5–9.5, with an optimum at 7.5–8.0. The composting conditions in our experiments were similar to these ranges, while *Tepidimicrobium* and *Caldibacillus* were represented a significant share of the FW community. Noteworthy, members of the order *Clostridiales* were previously identified in FW subjected to pretreatment under strict anaerobic conditions (Fisgativa et al., 2018). These results indicate that the microbial community of FW inoculated with composition **B2** was oxygen-deficient for at least the first 14 days. Anaerobic conditions can develop even within very small particles, since oxygen has a limited capacity of transferring from the gaseous to the liquid phase (Reinhardt, 2002). The presence of facultative anaerobic bacteria of the genera *Lactobacillus* and *Anoxybacillus* in substrate **B2** on day 15 of composting also supports this notion.

Limitations: Firstly, we used one aeration rate and did not increase it when using inoculum, which could affect the oxygen availability. Secondly, we used one FW moisture value, which was consistent with real values but higher than the recommended optimum values for composting (60 wt%). Thirdly, the cultures were washed from the medium, thus reducing the potential positive effect on composting of metabolic products accumulated during cultivation. However, our results are based on a large amount of experimental data from a significant number of parallel experiments on non-sterile raw FW of variable composition, which provides solid evidence for their use in composting acceleration.

To sum up, bacterial and fungal compositions from autochthonous cultures can be used together to intensify composting according to their growth characteristics. The composition of bacteria of the genus *Bacillus* is better introduced into the initial food waste – in a wetter and more rich in easily degradable organics substrate, and the composition of fungi of the genus *Penicillium* – at the cooling stage for degradation of difficult to degrade compounds.

The introduction of *Bacillus* at a low cell concentration of 4 × 10^5^ CFU g^−1^ of FW caused a decrease in the biodegradation rate, which was attributed to the difficulty in overcoming competition with the native waste microbial community.

The use of *Bacillus* inoculum with a concentration of 10^8^ CFU g^−1^ FW may be an effective way to increase the degradation rate of raw FW at moderate composting temperatures. A high titer of *Bacillus* inoculum during composting reduces oxygen availability and changes the degradation scenario of FW toward a higher proportion of anaerobic metabolism. This should be taken into account when using such composts and create conditions where the oxygen diffusion rate is more intense than the microbial activity. This may also be interesting to use as a process preceding anaerobic fermentation and increasing its efficiency, given that hydrolysis may be the limiting stage of fermentation.

Thus, this study expands the understanding of the influence of inoculation parameters and composition of hydrolytically active microbial compositions on the biodegradation of FW during composting. The results can be used for targeted design of microbial compositions.

## Data Availability

The datasets presented in this study can be found in NCBI repository under the following accession numbers: *Bacillus subtilis* BS2022 (PRJNA979896); *B. inaquosorum* GMPCOW1 (OR166016); *B. spizizenii* GMPCOW4 (OR187152); *B. xiamenensis* GMPCOW3 (OR187099); *B. velezensis* GMPCOW2 (OR185556); *B. amyloliquefaciens* BAM2022 (PRJNA979896); *Penicillium* sp. strain GMPCOW5 (OR187303); *Penicillium* sp. strain GMPCOW6 (OR187306); *P. crustosum*+*P. chrysogenum* GMPCOWM1 (PRJNA997142).
