# Supplementary material for: Enhancing aerobic composting of food waste by adding hydrolytically active microorganisms
Source: Front Microbiol. 2024 Dec 2;15:1487165. doi: 10.3389/fmicb.2024.1487165 (PMC11647035; doi:10.3389/fmicb.2024.1487165)
Supplement: Supplementary file 1 [file Supplementary_file_1.docx]

Supplementary Material

**Enhancing Aerobic Composting of Food Waste by adding Hydrolytically Active Microorganisms**

**Vladimir Mironov^1*^, Vitaly Zhukov^1^, Kristina Efremova^1^ and William F Brinton^2^**

^1^ Winogradsky Institute of Microbiology, Federal Research Center of Biotechnology, Russian Academy of Sciences, Moscow, Russia

^2^ Woods End Agricultural Institute Inc., Mount Vernon, Maine, USA

*** Correspondence:**Corresponding Author
7390530@gmail.com


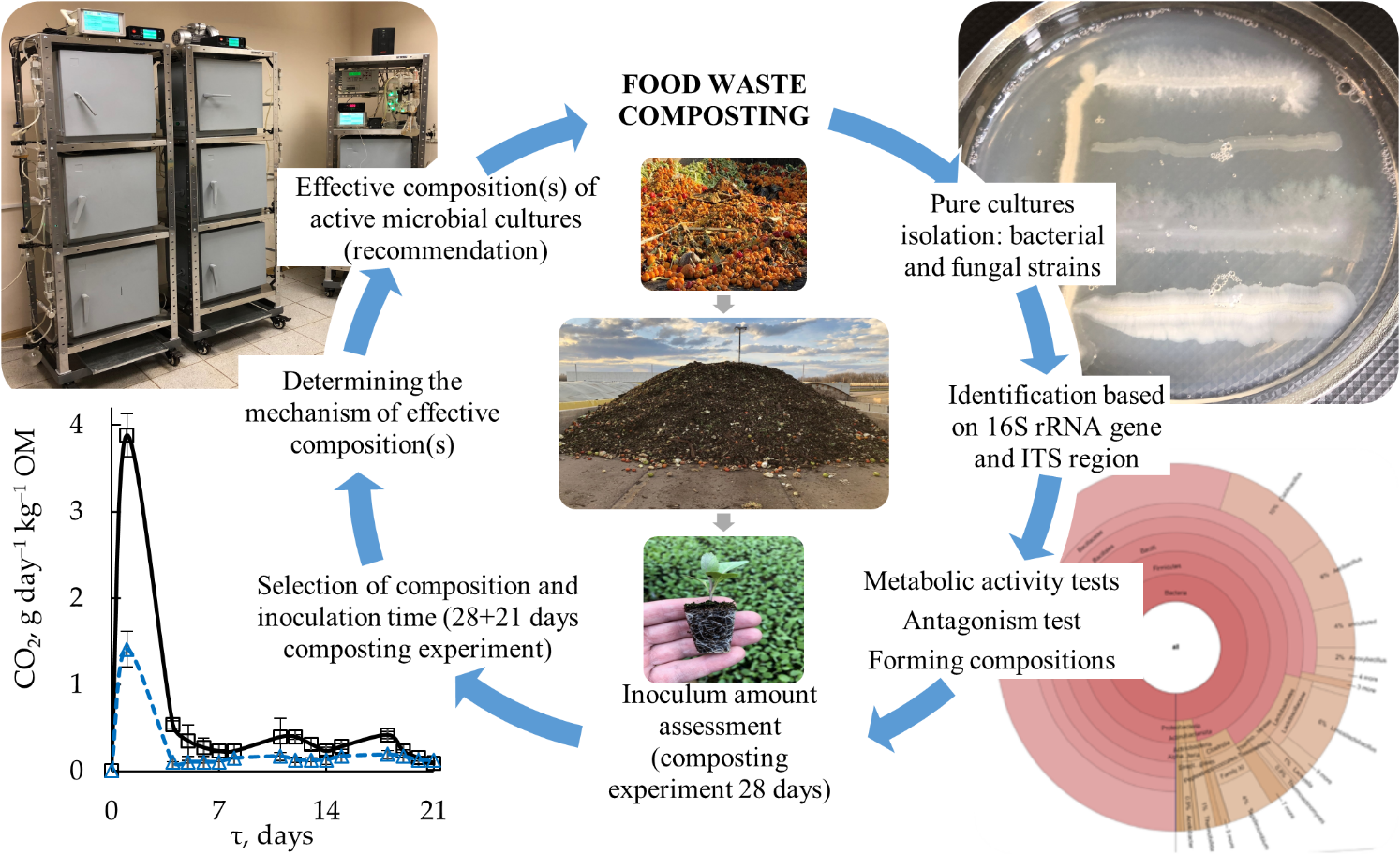


**Supplementary Figure 1.** General research design.

**
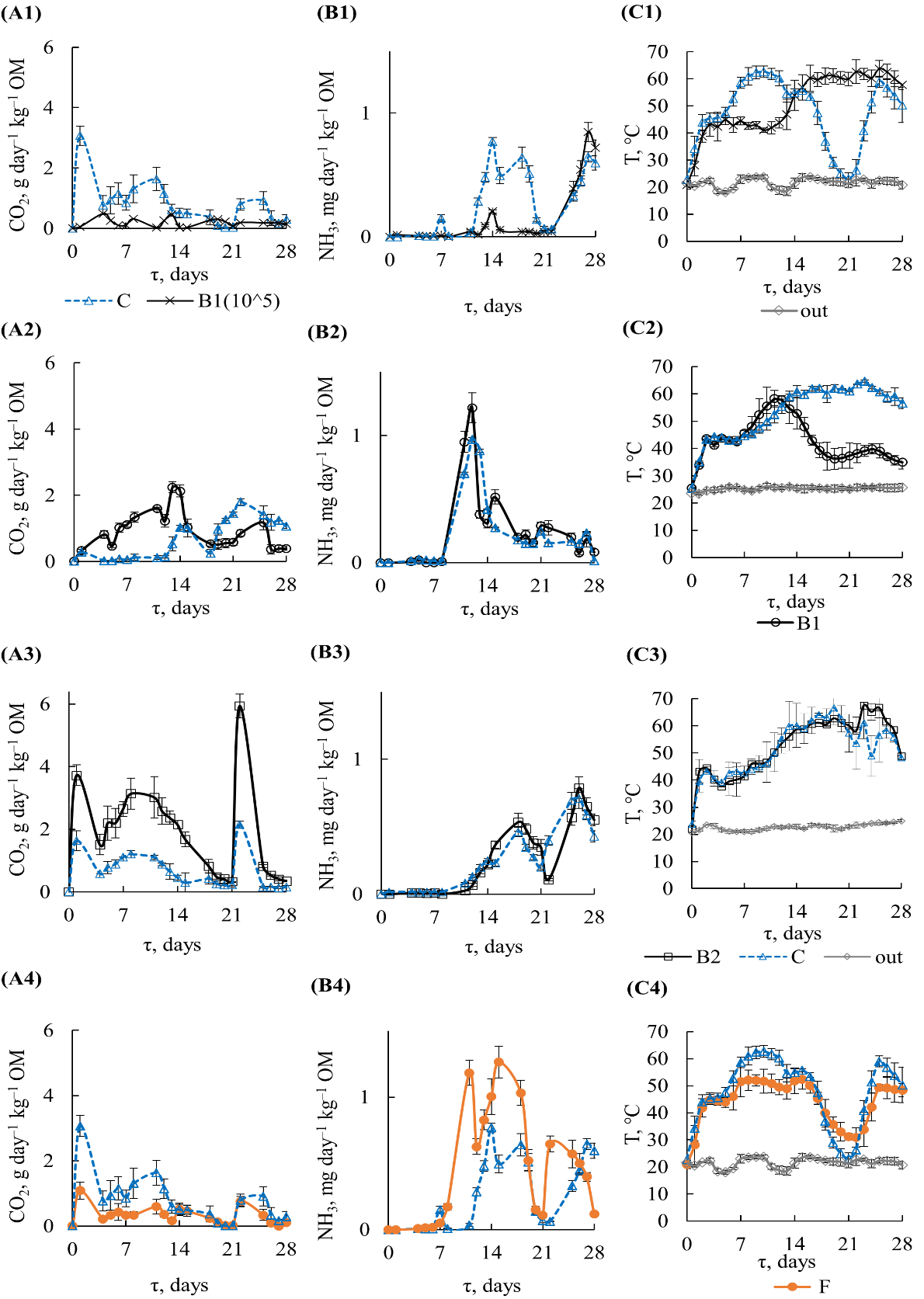
**

**Supplementary Figure 2.** START: dynamic profiles of (A) CO_2_, (B) NH_3_ and (C) temperature: 1 - composting with inoculation **B1** with concentration 4×10^5^ CFU g^–1^; 2 – **B1** with concentration 10^8^ CFU g^–1^; 3 – **B2** with concentration 10^8^ CFU g^–1^; 4 – **F** with concentration 10^4^ CFU g^−1^; C – control without inoculum; out – outside temperature.


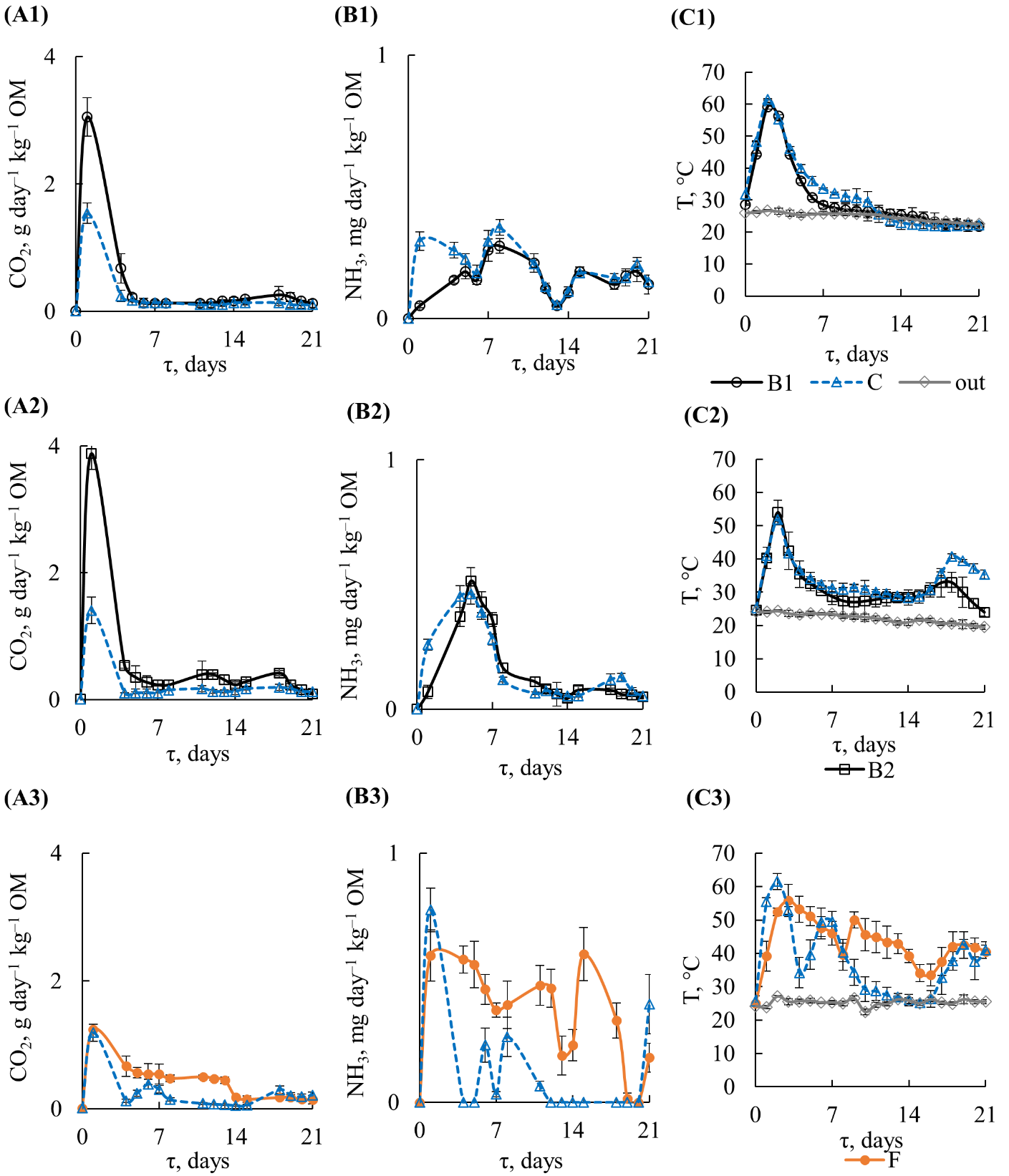


**Supplementary Figure 3.** RESTART: dynamic profiles of (A) CO_2_, (B) NH_3_ and (C) temperature: 1 – composting with inoculation **B1** with concentration 10^8^ CFU g^–1^; 2 – **B2** with concentration 10^8^ CFU g^–1^; 3 – **F** with concentration 10^4^ CFU g^−1^; C – control without inoculum; out – outside temperature.

**Supplementary Table 1.** Comparative evaluation of the effect of inoculation during composting

| **Microorganisms** | **Characteristics** | **Titre** | **Time of inoculation** | **Substrate** | **Result** | **References** |
| --- | --- | --- | --- | --- | --- | --- |
| *Bacillus subtilis,*  *Bacillus licheniformis, Phanerochaete chrysosporium, Trichoderma koningii, Saccharomyces cerevisiae,*  *Bacillus amyloliquefaciens* | Commercial microbial agents | n.d.^*^ (1% on a raw weight basis) | At the start of the process (at forced heating up to 50 °C) | FW and corn straw | Increase in temperature and pH  Significant impact on the succession of the bacterial community | Wang et al. (2024) |
| *Dysgonomonas sp., Pseudomonas caeni, Aeribacillus pallidus, Pseudomonas sp., Lactobacillus salivarius, Bacillus thuringiensis, Bacillus cereus* | Anti-acid microbial consortium | ∼10^8^ CFU g^−1^ dry matter (DM) | At the start of the process | FW and wheat bran | Accelerates the breakdown of carbohydrates  Increases the diversity of microbes producing key enzymes in the acetic and propionic acid metabolic pathways, as well as the number of these key enzymes | Song et al. (2018) |
| *Brevibacillus borstelensis*,  *Bacillus licheniformis*,  *Bacillus thuringiensis*,  *Bacillus cereus* | A consortium of thermophilic proteolytic, amylolytic, cellulolytic and oil-degrading bacteria | n.d.  (6.3×10^8^ CFU mL^−1^ nutrient broth) | At the start of the process | FW and sawdust (4:1 dry weight) | The thermophilic stage came on faster by 2 weeks  Maintaining a higher pH level  High CO_2_ production (133.94 vs. 107.75 g CO_2_ loss/kg composting mass) | Awasthi et al. (2017) |
| *Brevibacillus borstelensis*, *Bacillus cereus*, *Bacillus licheniformis*,  *Brevibacillus agri* | A consortium of thermophilic oil degrading bacteria | n.d.  (6.9×10^7^ CFU mL^−1^ nutrient broth) | At the start of the process | FW and sawdust (4:1 dry weight) | The thermophilic stage came on faster by 2 weeks  Maintaining a higher pH level  High CO_2_ production (116.50 vs. 107.75 g CO_2_ loss/kg composting mass) | Awasthi et al. (2017) |
| *Thermoactinomyces vulgaris* A31 | Thermo-tolerant lipolytic actinomycete (primary inoculum) | ∼10^5^ CFU g^−1^ DM | At the start of the process | FW with high fat content (primary inoculum /FW/ sawdust at a ratio of 2:10:1) | Decrease in fat content by 7%  Increase in CO_2_ emission in the first 10 days with maximum difference from ∼15 to ∼23 mg CO_2_-C g^−1^ compost-C d^−1^  Increase in titre on day 16 to ∼10^8^ CFU g^−1^ DM | Ke et al. (2010) |
| *Pichia kudriavzevii* RB1 | Mesophilic the acid-consuming yeast | 10^7^ CFU g^−1^ raw weight | At the start of the process (with temperature control at 40 °C) | Model FW (rabbit feed and rice) and sawdust | Increased decomposition of acetic acid and increased activity of waste bacteria  The maximum CO_2_ release was 2 days earlier than in the control | Nakasaki et al. (2013),  Nakasaki, Hirai (2017) |
| *Bacillus* (7%),  *Bacteroides* (6%),  *Mycobacterium* (5%),  *Methylomonas* (5%)  and others | A complex microbial agent containing more than 20 different microorganisms | 10^8^ CFU g^−1^ raw weight | In two stages: at the start of the process and when the temperature drops (on day 19) | FW and rice husk | Increased microbial interaction by 1.2 times  Increased time of maintaining high composting temperature by 33%  Increase of humic acid content by 42% and obtaining high germination index (101.69%) | Zhu et al. (2023) |
| *Bacillus safensis* YM1 | Salt-resistant and oil-degrading | ∼10^7^ CFU g^−1^ raw weight | At the start of the process | FW and sawdust | Temperatures above 50 °C were 3 days longer Ammonification increased during the cooling phase. Oil content and the salt (Cl^−^) concentration decreased significantly by 19.7% and 8.1%, respectively  GI 84.8% against 72.1% in the control | Zhang et al. (2024) |
| The compound microbial agents | Photosynthetic bacteria, yeast, bacillus and lactic acid bacteria | ∼10^7^ CFU g^−1^ DM | At the start of the process | FW and rice bran | CO_2_ reduction by 20-30% | Xie et al. (2023) |
| *Bacillus* (*B. subtilis*, *B. tequilensis*, *B. venezuelans и B. amyloliquefaciens*) | Cellulolytic bacterial inoculum obtained from landfill soil | ∼10^6^ CFU g^−1^ raw weight | At the start of the process | OFMSW | Increase in maximum temperature to 46 °C vs. 39 °C for the control  Decrease in C/N to 16.4 vs. 17.2 for control | Rastogi et al. (2019) |
| Trametes versicolor,  Fomes fomentarius | The white-rot fungi | n.d. (10 g suspension mixture kg^−1^ raw weight) | At the end of the thermophilic stage (after 37 days) | OFMSW | Higher decomposition rate and better maturity rate | Voběrková et al. (2017) |
| Bacillus thuringiensis | Commercial strain of *Bacillus thuringiensis* subspecies *kurstaki* | Inoculation at the beginning: 5.8×10^6^ CFU g^−1^ DM  Inoculation at 40 °C: 2.5×10^6^ CFU g^−1^ DM | At the start of the process or at the end of the thermophilic stage (temperature around 40 °C) | OFMSW with wood chips (1:1) | Inoculation at the start of the process: the number of viable cells decreased to 3×10^5^ CFU g^−1^ DM by day 15 with a further increase to ∼10^6^ CFU g^−1^ DM by day 28  Inoculation at the end of the thermophilic stage: increase to 9×10^6^ CFU g^−1^ DM after 30 days | Ballardo et al. (2017) |
| Bacterial and bacterial-fungal compositions | Bacterial composition: ammonia-oxidising, nitrifying and deodorising bacteria   - Bacterial-fungal compositions: cellulolytic and lignolytics | 10^5^ CFU g^–1^ DM | At the start of the process or multi-stage | OFMSW with dry grass | The multi-stage inoculation method prolonged the high temperature period and effectively increased the secondary fermentation temperature, improved the community structure and functional diversity of bacteria and fungi | Xi et al. (2015) |
| *Trichoderma viride*, *Aspergillus niger*,  *Aspergillus flavus* | Selection of the strains was made on the basis of synergistic enzymatic activity (cellualses, protease, amylase and lipases) | ∼10^5^ CFU g^−1^ DM | Inoculation after 3 days of composting | OFMSW | The time to the start of the thermophilic phase is reduced  Loss of organic matter increased  Increased pH | Awasthi et al. (2014) |
| *Bacillus subtilis, Bacillus amyloliquefaciens* (**В2**) | Hydrolithics | 10^8^ CFU g^–1^ raw weight | At the start of the process | FW | Increased CO_2_ formation by 2.8 times while keeping ammonia formation and temperature the same  10 wt% more OM loss  Increase GI to 128% on day 28 | data from this work |
| *Penicillium* sp. (**F**) | Hydrolithics | 10^4^ CFU g^−1^ raw weight | On the 28th day, when the temperature goes down | FW | Increased CO_2_ formation by 1.8 times, temperature by 5.6 °C and ammonia emission by 3.1 times | data from this work |

^*^ n.d. - no data
